# Development and validation of an intervention for childhood trauma and exposure to violence in Vhembe District, South Africa

**DOI:** 10.1016/j.mex.2024.102651

**Published:** 2024-03-18

**Authors:** Petunia Tsheole, Lufuno Makhado, Angelina Maphula, Nombulelo Veronica Sepeng

**Affiliations:** a University of Venda, Private Bag X5050, Thoyandou, Limpopo 0950, South Africa; b Faculty of Health Sciences, University of Pretoria, Bophelo Road, Prinshof 348-JR, Tshwane, Pretoria 0082, South Africa

**Keywords:** Child trauma, Interventions, Exposure to violence, Multi-Phases, SOP framework, South Africa, Donabedian structure, process and outcome model (SPO).

## Abstract

Most strategies are implemented; however, South Africa needs to evaluate and develop trauma interventions. The study aims to develop, test and validate childhood trauma exposure intervention in the Vhembe district, Limpopo province. Donabedian's structure-process-outcome model will guide the study. The study will employ multiphase mixed methods with five phases. Phase 1 will be a thorough systematic evaluation of literature on childhood trauma and exposure to violence interventions to describe existing interventions. Phase 2, stage 1: Will explore the experiences of children exposed to trauma and violence regarding their experiences of the treatment they received, using semi-structured qualitative interviews. Non-probability purposeful sampling techniques will be used to select participants. The Thoyondou Victim Empowerment's database will select participants. The researchers will conduct semi-structured and unstructured interviews with youngsters exposed to violence and trauma. Stage 2 will be a qualitative study of trauma centre managers and personnel sampled from the contact record. IPA will analyze data. Phase 3 will conceptualize Phase 1 and the empirical phase into Donabedian's SPO framework for Phase 4. Phase 4 develops the intervention using Phase 3′s conceptual framework and tests and validates it.

## Graphical abstract

**Figure:**
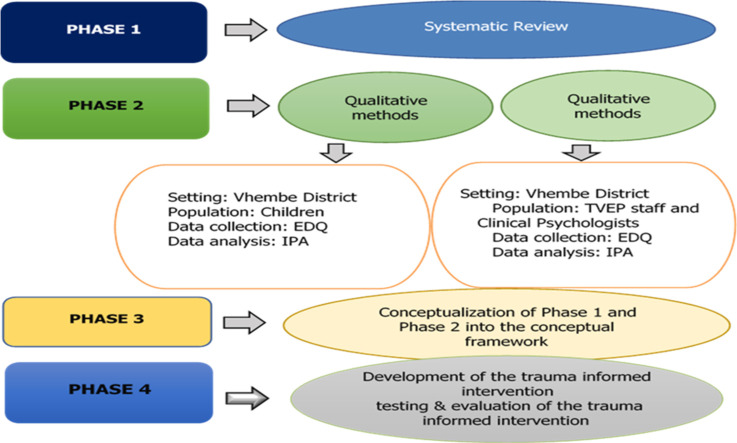

Specifications table

**Table:** 

Subject area:	Psychology
More specific subject area:	Child trauma
Name of your protocol:	Development and validation of an intervention for childhood trauma and exposure to violence in Vhembe District, South Africa.
Reagents/tools:	Not applicable
Experimental design:	Not applicable
Trial registration	SHS/21/PSYCH/08/2411
Ethics:	Written informed consent and assent from parents and children will be sought before data collection.
Value of the Protocol:	• Development of Childhood trauma and exposure to Violence intervention, test and validate an intervention in Vhembe district, Limpopo province. • This study will adopt the (6SQuID) model that follows six steps in quality intervention • Development of the intervention to offer quality trauma-informed care with the TVEP facilities following a step-by-step process.

## Description of protocol

### Background information

Childhood trauma and exposure to violence is a global problem, with the problem ranging from children modelling behaviours of violence, perpetrating violence on other children and experiencing mental challenges. This study seeks to explore, develop, test and validate an intervention in the Vhembe district, Limpopo province.

### Study rationale

The potential outcome of this research study may add value to the already existing body of literature about the management of childhood trauma and exposure to violence on children within a rural setting and a tailor-made intervention. The developed intervention will be utilized by other professionals who work directly with children, aiming to manage the trauma symptoms and waiting time for consultations with psychologists and mitigate the occurrence of long-term effects. Furthermore, there is potential to inform policymakers about the importance of early trauma intervention among children to prevent adverse effects and improve the quality of life and well-being of children.

### Study purpose and objectives

The following are the study's purpose and objectives.

### Study purpose

The study aims to develop, test and validate childhood trauma exposure intervention in the Vhembe district, Limpopo province.

### Study objectives

#### Phase 1: systematic review

1. To establish available childhood trauma and exposure to violence interventions and their effectiveness.

#### Phase 2: empirical phase

Stage 1: Qualitative study

1. To explore and describe the experiences of children regarding the management of childhood trauma and violence exposure in the Vhembe district.

Stage 2: Qualitative study

1. To explore and describe the perspective of professionals regarding the available childhood trauma and violence exposure interventions employed in the Vhembe district.
2. To explore and describe the barriers experienced by mental health care professionals in managing childhood trauma and violence exposure among children.

#### Phase 3: conceptualization of findings into a conceptual framework

1. To conceptualize the findings from Phase 1 and Phase 2 into the conceptual framework.

#### Phase 4: development of the trauma-informed intervention testing and evaluation of the trauma-informed intervention

1. To develop trauma intervention for children exposed to violence and traumatic situations.
2. To test and validate the effectiveness of the trauma intervention developed for children exposed to trauma and violence.

Research questions

1. What are the available childhood trauma and exposure to violence interventions?
2. What are the experiences of children regarding the management of childhood trauma and exposure to violence in the Vhembe district?
3. What are the experiences of children regarding the management of childhood trauma and violence exposure in the Vhembe district?
4. What are the interventions in place to assess and treat children exposed to violence and traumatic situations?
5. What are the barriers and facilitators in managing childhood trauma and violence exposure in the Vhembe district?
6. What are the stages involved in the development of trauma intervention and exposure to violence interventions for children who have experienced trauma and exposure to violence?
7. What are the monitoring and evaluation tools used to measure the effectiveness and validity of child trauma intervention and exposure to violence?

### Conceptual framework of the study

The SPO model recommends that healthcare quality concerning trauma be evaluated using the following: the structure will refer to (characteristics of the healthcare facility setting), the process (clinical processes performed in the healthcare facility setting), and outcome (ultimate status of the patient following trauma-informed care interventions). According to this model, improvements in the structure of care in TVEP facilities will improve clinical processes and patient outcomes. Donabedian structure process model is the more flexible framework to be applied in this study. It will serve as a framework to analyse the underlying mechanisms contributing to trauma management [1].

### Definition of concepts

**Child:** According to South African law, a child means a person under the age of 18 years [2]. For this research study, a child is a person aged 6–17 years.

**Trauma:** According to the APA, trauma is defined as any distressing experience, such as rape, war and or witnessing a murder, that brings about significant fear, confusion, and disruptive feeling intense enough to have a long-lasting negative effect. These negative effects range from negative behaviour and attitudes and affect other aspects of an individual [3]. In this research study, trauma will be conceptualized as a significant event that transpired to a child or witnessed by a child and has caused significant emotional distress and mental effects resulting in fear, helplessness, behavioural problems and emotional dysregulation.

**Violence:** Violence is an act, whether direct or indirect, used to injure or abuse a person, group or community [4]. In this study, violence will be conceptualized as an act that is intended to cause harm to a child or a community to which a child belongs.

## Methodology

The study will follow a mixed multi-phased qualitative method.

### Research approach

The researcher will begin by first conducting a Phase 1 systematic review of the literature relating to childhood trauma and exposure to violence interventions in an attempt to gather enough information to describe available strategies that are currently used. Phase 2 will be the empirical phase comprising Stage 1: Qualitative methods will explore the types of trauma and the effects of trauma on the child. In contrast, Stage 2: Qualitative method will focus on the TVEP manager and health care workers as well as clinical psychologists within the Vhembe district to explore the available childhood trauma and violence exposure interventions employed in the Vhembe district and barriers and facilitators in managing childhood trauma and violence exposure among children. Subsequently, Phase 3 will comprise the findings of Phase 1 and Phase 2 conceptualized into the SPO conceptual framework. Phase 4 aims to develop, test and validate childhood trauma and violence intervention guided by the consolidated and conceptualized conceptual framework of Phase 3.

### Phase 1

This phase will comprise a systematic review. The systematic review will target the interventions and their effectiveness published globally from 01 January 2011 until 31 July 2023 on childhood trauma exposure and violence. The interventions and their effectiveness may be published in peer-reviewed journals or original interventions published on websites such as the World Health Organization. Different search engines such as Science Direct, Google Scholar, EBSCOhost, PubMed and PsycINFO databases will be used to surf through the appropriateness of the literature. These databases are the most relevant choices for studying interventions and their effectiveness concerning childhood trauma exposure and violence due to their comprehensive coverage, interdisciplinary nature, quality assurance, diverse sources, currency, and depth of search capabilities, collectively supporting a robust and informed exploration of the topic. Inclusion criteria will be articles that focus on the treatment of children exposed to trauma and violence. Exclusion criteria will be articles that do not contain information about interventions used to treat children exposed to trauma and violence.

### Design

A systematic review will be done. This is to identify available trauma and trauma-focused interventions in a community setting and assist relevant facilitators in improving clinical outcomes. Furthermore, this review will inform a research agenda on implementing trauma-focused interventions for children by summarizing current knowledge of interventions. (*Review title*: Childhood trauma and exposure to violence interventions: The need for effective and feasible evidence-based interventions: A systematic review)

*Review questions*

1. What are the available childhood trauma and exposure to violence interventions?

*Specific objective*

1. To establish available childhood trauma and exposure to violence interventions and their effectiveness

### Search strategy for identification of studies

Different search engines such as Science Direct, Google Scholar, EBSCOhost, PubMed and PsycINFO databases will be used to surf through the appropriateness of the literature. The inquiry was conducted via various internationally recognized databases to collect relevant information from published sources. Science Direct is an online digital library that serves as a repository for intellectual scientific research. It is overseen and administered by Elsevier, a highly regarded publisher in the academic community. The platform functions as an internet-based scholarly citation index, enabling users to access a diverse array of published scientific literature conveniently. PubMed and other scholarly databases function as supplementary tools that enable discovering and retrieving academic content to enhance the healthcare domain. Google Scholar lacks a comprehensive compilation of publishers, journals, types of journals, and information about the temporal scope of the peer-reviewed status of its entries. Utilizing a sophisticated search engine within Google Scholar significantly benefits capturing citations and duplicates that other databases may not cover. Psych info provides a complete perspective on the behavioural and social sciences, serving as a well-established repository of research material and findings essential for building a comprehensive collection in psychology.

### Inclusion criteria

Inclusion criteria will focus on articles that are published on interventions addressing childhood trauma and exposure to violence. The interventions should be used to manage children from the age of 6 years to 17 years. The interventions should be published in peer-reviewed journals globally or on websites such as World Health Organizations from 01 January 2011 until 31 July 2023. The papers will be searched and accessed on Science Direct, Google Scholar, EBSCO—Host, PubMed and Psych-info databases.

### Exclusion criteria

The review will not consider articles that solely address sexual abuse or treat it as a separate type of trauma. Additionally, studies primarily focused on clinical disorders like depression or anxiety without any link to trauma history will be excluded. Articles that exclusively focus on children with behavioural problems without considering trauma, those conducted outside of healthcare contexts, or those not involving healthcare practitioners will also be omitted from the review.

### Assessing the quality of articles

The researchers will use a structured checklist called the Critical Appraisal Skills Programme (CASP) to assess article quality for inclusion in the review. This checklist has ten questions, grouped into three parts: credibility, relevance, and findings. Each question gets a ``yes,'' ``unclear,'' or ``no'' answer. To score each article, the researchers will independently assign points from 0 to 10. These points depend on how many ``yes'' answers we get during the checklist evaluation. The researchers will then discuss and reach a consensus, and articles with more than five ``yes'' responses distributed across the credibility, relevance, and findings will be included for the review.

### Presentation of results

This study's findings will be summarized in the table. The purpose of summarizing the findings in a table is to draw themes on interventions addressing childhood trauma and violence exposure, effective interventions managing childhood trauma and violence exposure and their feasibility, as well as interventions that are not effective in managing childhood trauma and violence exposure.

### Phase 2

Phase 2 stage will focus on the organizational context that relates to patient recovery issues. The rationale for using the Qualitative method in this phase of the study is to understand the day-to-day responsibilities of the facilitators in Thoyondou Victim Empowerment. Interviewing facilitators will give an in-depth understanding of the context's perceived influences on transition decisions and associated challenges experienced and patient outcomes issues.

### Stage 1: qualitative study design

The study design of stage 1 will be qualitative exploratory, descriptive methods. According to Stebbins [5], exploratory research aims to provide insights into and an understanding of the problem identified by the researcher. Previous studies suggest that descriptive research, on the other hand, aims at describing something, mainly functions and characteristics [6]. Qualitative research focuses on obtaining relevant, comprehensive and specific information about a phenomenon in a natural environment [7].

### Study setting

Phase 1 of the project would involve conducting a comprehensive systematic review to gather literature from various sources worldwide. The study for stage 1 in phase 2 will be conducted in TVEP centres in Thulamela Municipality in the Vhembe district. This will enable the participants to be comfortable in their natural setting and enable the researcher to observe the participants while interviewing them.

### Study population and sampling

*The target population for stage 1 in phase 2:* Children aged 9–17 in Thulamela municipality Vhembe district in Thoyondou Victim Empowerment facility. The rationale for selecting children within this age range for sampling is based on their capacity to articulate a comprehensive narrative of the trauma intervention they have undergone. This is done to gain a deeper understanding and awareness of the various types of trauma they have encountered, as well as to comprehend how trauma-informed therapies have assisted them in navigating their unique experiences.

### Sample and sampling

Non-probability purposive sampling will be used to select children who have been exposed to domestic violence and childhood trauma aged between 9 and 17 years. A homogeneous sample from the TVEP centre will hold some relevance and personal significance to respondents and enable researchers to capture details on a specific group of participants who have experienced a particular phenomenon. Data saturation will determine the sample size for the study phase. Data saturation refers to the point in a research study where researchers have gathered sufficient information, such that any more data collected does not yield new insights or knowledge [8].

### Data collection

In this stage, semi-structured interviews will be conducted with children exposed to violence and traumatic situations. The researcher will recruit participants from the existing database at TVEP facilities in the Vhembe district. Rapport will be built from the first contact meeting with the children and their parents or guardians, where the study topic, problem, and purpose will be discussed to prepare for data collection day and to build a good relationship. The room used for data collection will be prepared beforehand, the place will be free from any disturbances, and privacy will be ensured.

### Data analysis

Interpretative phenomenological analysis (IPA) will be used for data analysis in this phase. IPA is chosen as a method of data analysis because, according to Noon and Hallam, analysis for IPA goes beyond thematic analysis, furthermore, it is multidirectional and allows the researcher to adapt it following the objectives [9].

**Table:** 

Reading and note-making	Recorded interviews will be transcribed; the transcribed data will be read and re-read while listening to the audio recording to confirm the transcribed data. This will ensure that a deeper and interpretative analysis is produced.
Notes to emerging themes	Once the researcher has reviewed the transcript, the emerging themes will be noted and documented to create connections.
Connecting emergence themes	Referrals will be made to the transcripts to ensure the connections are consistent with the raw data. The essence of this stage is to ensure that the researcher stays within the participants' exact words.
Producing table for themes	Emerging themes will be organized into a table and sub-themes within their respective subordinate themes, along with the retrieved relevant receptive theme extracted from the transcribed data. A page number will also be included to enable them to retain the participant's personal experience.
Final table	The data transcribed with themes and subthemes will be organized into a table. This will enable the researcher to decide which themes should be prioritized and which ones to be abandoned. Prioritizing of themes will be given to more articulate participants

### Stage 2: qualitative study design

The study design of stage 2 of phase 2 will be qualitative exploratory, descriptive methods.

### Study setting

The study setting will be in the Vhembe district. Multiple sites will be utilized, such as TVEP facilities, private practices, clinics or hospitals where trauma-related mental health providers are available. This will enable the participants to be comfortable in their natural setting and enable the researchers to observe them while interviewing them for observational notes.

### Study population and sampling

*The* study population will be trauma center's health care employees and clinical psychologists will be sampled using total population sampling by approaching the Health Professional Council of South Africa for the list of psychologists currently registered with the professional body and based in Vhembe district, Limpopo province. The rationale for using the psychologists and TVEP employees is to explore their experiences on the interventions used to treat trauma amongst children and possible barriers experienced with the existing treatment interventions they have used.

### Sample and sampling

This study will sample all the trauma centre's employees, managers, and health care providers (clinical psychologists, registered trauma counsellors), both in the public and private sectors. The sample size will be determined by data saturation. Data saturation refers to the point in a research study where researchers have gathered sufficient information, such that any more data collected does not yield new insights or knowledge [8].

### Data collection

Data collection will be conducted using semi-structured interviews among TVEP staff members and clinical psychologists working with children, offering a service to children exposed to trauma and violence. Interviews with TVEP staff members will provide an overview of the organization and service delivery.

### Data analysis

Interpretative phenomenological analysis (IPA) following the steps in stage 1 of phase 2 will be used to analyze data in stage 2 of phase 2.

### Trustworthiness credibility

The trustworthiness of the findings will be verified by performing this research under the direction of professionals with expertise in designing and validating interventions. This study was conducted by professionals with expertise in intervention creation and validation to guarantee its credibility.

### Transferability

Transferability in qualitative research will ensure that the research provides a clear and in-depth description. Furthermore, purposeful sampling will ensure that the data captured is from relevant participants. The research technique that will be used to develop and validate the intervention is outlined in detail to ensure that the program is transferable. This allows the program to be transferred or utilized in other settings or other provinces of South Africa.

### Dependability

Dependability in qualitative research refers to the stability of data over time and the conditions of the study.

The research methodology followed to develop and validate child trauma and exposure to violence intervention will be described in detail as a way of ensuring dependability.

### Confirmability

Confirmability will be accomplished through the use of recording tape and field notes. This will be done to ensure that the data accurately represents the information the participants provided.

### Phase 3 conceptualization of phase 1 and 2 findings into a conceptual framework

**Fig. 1:**
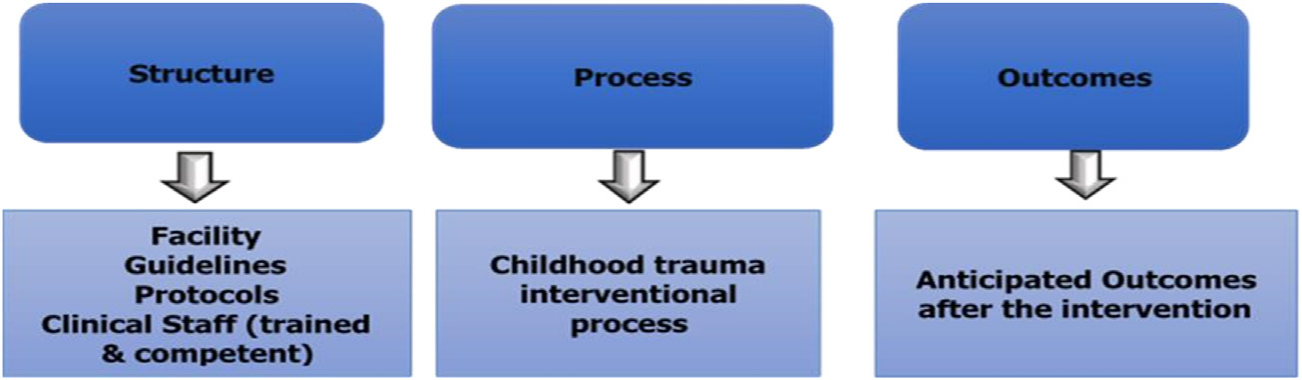
The Donabedian structure process model [1].

In this phase, the researcher will conceptualize the findings for phases 1 and 2 into the Structure-Process -Outcome (SPO) model. The structure will be characteristics of the healthcare facility setting, the process will be clinical processes performed in the healthcare facility setting, and the outcome will be the ultimate status of the patient following trauma-informed care interventions. See Fig. 1.

### Phase 4 development, testing and evaluation of the trauma-informed intervention

#### Design

**Fig. 2:**
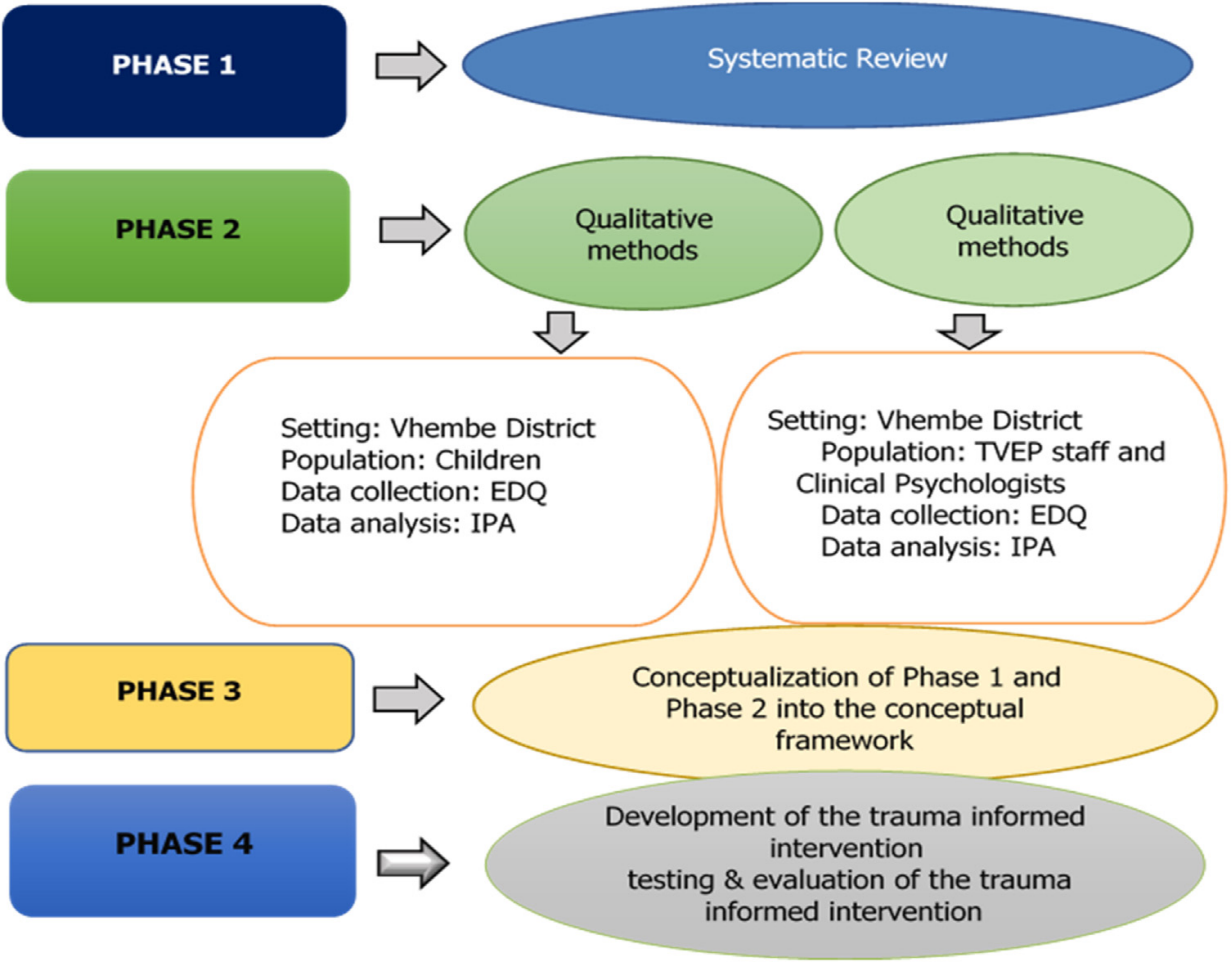
Research strategy (Researchers’ own work).

In this phase, the researchers will use the conceptualized findings in phase 3 to develop and test the intervention. To achieve that, the researchers will follow Wight et al.’s six steps to develop an intervention [10]. The first step is defining and understanding the problem and its causes [10]. This study will conduct individual interviews to understand the problems and their causes. The second step is identifying modifiable causal or contextual factors with the greatest scope for change and who would benefit most. In this study, the researchers will identify modifiable causes or contextual factors, such as techniques and activities. The third step is deciding on the mechanisms of change. Thus, the information acquired from the interview sessions will decide the mechanisms of change. The fourth step is clarifying how these will be delivered. This study will deliver the mechanisms for change through a hard copy as an intervention for change. The fifth step is testing and adapting the intervention. In this study, the last step is for the researcher to collect sufficient evidence of effectiveness to ensure the rigorous evaluation/implementation of the trauma intervention collecting sufficient evidence of effectiveness to proceed to a rigorous evaluation. To ensure the intervention's rigour, the following steps will be taken to ensure usability and adaptability. In this study Fig. 2 below is summarizing the overall research strategy that will be followed in this study.

### Ethical consideration

The following ethical considerations will be considered in the study.

### Ethical clearance

The study proposal and tools were submitted to the University of Venda Human Research Ethics Committee for ethical clearance [SHS/21/PSYCH/08/2411].

### Ethics statements

Ethical clearance will be sought from the University of Venda Human and Clinical Trails Research Ethics (HCTREC) before the commencement of the research project. Permission to conduct the study in selected schools will be sought from the Department of Basic Education. Consent forms for the TVEP facilitators and psychologists will be distributed. The participants can terminate their agreement to participate at any time during the interviews. Written informed consent from parents and assent from children will be sought before data collection.

### Anonymity

Names and the identifying particulars of participants will be protected by assigning Pseudo-names. The researcher will ensure that data is not linked to any participant. Caution will be used, and participants will be identified using numerical digits or alphabetical letters.

### Confidentiality privacy

The researcher will treat all the private information collected about the participants as highly confidential. The collected data, audio recordings, and field notes will be stored in a locked room with good ventilation [[10], [11]]. The principal researcher, supervisor and co-supervisor will be the only people accessing the collected data. Should the researcher involve facilitators, discussion and signing confidentiality and privacy forms will be made available to them to ensure that they are aware of the obligation of not discussing the content or identifying particulars of the participants.

### Beneficence

This principle covers the right to freedom from any harmful treatment. In this study, the researcher will prevent harm to participants and maximize the benefits of participating in this study. This principle will be followed by complying with respect for human dignity, justice, objectivity and integrity in research.

### Respect for human dignity

This principle emphasizes that all the participants must be treated as human beings to respect their human dignity. These principles will ensure that the researcher and participants respect the right to human dignity, the right to full disclosure, and the right to self-determination [12].

### Justice

Justice in research underscores fairness and privacy for study participants. This means that participants have the right to be treated fairly and to remain anonymous during the study.

### Objectivity and integrity in research

The researcher will always seek to maintain objectivity and honesty in conducting research. All limitations of the findings that may compromise their validity will be disclosed. In doing so, the researcher will not alter any collected data to affect the trajectory of the study.

### Delimitation of the study

The study will take place in all 3 sites of TVEP centres in the Vhembe district, and all professionals attached to the centres will be interviewed.

a. Conference presentations will be made to share the study results.
b. Presentation of study results will also be made during research days at the university.
c. The study's findings will be disseminated through presentations, focusing on healthcare systems that treat children with trauma. Both parents and children will be invited to these presentations

### Plan for dissemination and implementation of results

For this research protocol, the dissemination of findings will be done using the central research argument, which will be based on the outcomes of the research paper. The findings of this research report will be released or distributed to expand the existing body of scientific knowledge. The research report will be submitted to a professional journal and presented at academic conferences. Copies of the research report will be distributed to the TVEP facilities in Limpopo province and inform participants about research outcomes.

## CRediT author statement

**Ms. Petunia Tsheole:** Conceptualization, protocol design and development, Writing – original draft, editing, and revisions. **Prof. Lufuno Makhado:** Main research supervisor, Conceptualization, Protocol design and development supervision, guidance, feedback, editing, and revisions. **Prof. Angelina Maphula:** Co-supervisor, Writing – review & editing. **Dr Nombulelo Veronica Sepeng:** Writing – review & editing, and co-supervision.

## Declaration of competing interest

The authors declare that they have no known competing financial interests or personal relationships that could have appeared to influence the work reported in this paper.

## Data Availability

Data will be made available on request.
